# Is the *in vivo* dosimetry with the OneDosePlus^TM^ system able to detect intra-fraction motion? A retrospective analysis of *in vivo* data from breast and prostate patients

**DOI:** 10.1186/1748-717X-7-97

**Published:** 2012-06-20

**Authors:** Maria Daniela Falco, Marco D’Andrea, Alessia Lo Bosco, Mauro Rebuzzi, Elisabetta Ponti, Barbara Tolu, Grazia Tortorelli, Rosaria Barbarino, Luana Di Murro, Riccardo Santoni

**Affiliations:** 1 Department of Diagnostic Imaging, Molecular Imaging, Interventional Radiology and Radiotherapy, Tor Vergata University General Hospital, V.le Oxford 81, 00133, Rome, Italy; 2 Laboratory of Medical Physics and Expert Systems, National Cancer Institute Regina Elena, V. E. Chianesi 53, 00144, Rome, Italy; 3 Department of Physics, Tor Vergata University, V. della Ricerca Scientifica 1, 00133, Rome, Italy

**Keywords:** OneDosePlus^TM^ system, MOSFET detector, *In vivo* dosimetry, Intra-fraction motion error

## Abstract

**Background:**

The OneDosePlus^TM^ system, based on MOSFET solid-state radiation detectors and a handheld dosimetry reader, has been used to evaluate intra-fraction movements of patients with breast and prostate cancer.

**Methods:**

An Action Threshold (AT), defined as the maximum acceptable discrepancy between measured dose and dose calculated with the Treatment Planning System (TPS) (for each field) has been determined from phantom data. To investigate the sensitivity of the system to direction of the patient movements, fixed displacements have been simulated in phantom. The AT has been used as an indicator to establish if patients move during a treatment session, after having verified the set-up with 2D and/or 3D images. Phantom tests have been performed matching different linear accelerators and two TPSs (TPS1 and TPS2).

**Results:**

The ATs have been found to be very similar (5.0% for TPS1 and 4.5% for TPS2). From statistical data analysis, the system has been found not sensitive enough to reveal displacements smaller than 1 cm (within two standard deviations). The ATs applied to in vivo treatments showed that among the twenty five patients treated for breast cancer, only four of them moved during each measurement session. Splitting data into medial and lateral field, two patients have been found to move during all these sessions; the others, instead, moved only in the second part of the treatment. Patients with prostate cancer have behaved better than patients with breast cancer. Only two out of twenty five moved in each measurement session.

**Conclusions:**

The method described in the paper, easily implemented in the clinical practice, combines all the advantages of in vivo procedures using the OneDosePlus^TM^ system with the possibility of detecting intra-fraction patient movements.

## Background

In vivo dosimetry, recommended by various national and international organizations is a Quality Assurance tool to measure radiation dose delivered to patients during radiotherapy [1-5]. These measurements can be compared to the planned doses specified by the oncologist and calculated by the Treatment Planning System (TPS) for the target and critical organs (e.g. rectum or spinal cord). In this way set-up, calculation, motion or transcription errors, that may have gone unnoticed during pre-treatment check, can be recovered. In the absence of errors, routine in vivo dose measurements document that the treatment was delivered correctly.

Detectors commonly used for in vivo measurements are thermoluminescence dosimeters (TLDs), semiconductor diodes and Gafchromic® films (International Speciality Products, Wayne, NJ) [6,7]. All these devices have strong and weak points [8]; MOSFET detectors (Metal Oxide Silicon Field Effect Transistors) are a valid alternative as in vivo dosimeters [9-13]. They were designed to replace TLDs having about the same size and fewer correction factors as compared to diodes. However, like diodes, they have to be connected to commercial electrometers using cables, which can be discomforting for the patient. Since 2003, the OneDose^TM^ and since 2006 the OneDosePlus^TM^ systems (Sicel Technologies, Morrisville, NC), based on p-type MOSFET detectors, have been introduced to measure patient dose in radiotherapy [14-22]. Both systems have all the advantages of MOSFET detectors plus other interesting features. The dosimeters are wireless, precalibrated (the calibration factors for each dosimeter give the relationship between the voltage shift and the amount of radiation dose) and contain an adhesive backing to be attached to the patient. In the OneDose^TM^ system, the dosimeters for photon and electron beams are the same, and the user must provide a bolus to achieve the energy dependent build-up; in the OneDosePlus^TM^ system, instead, the dosimeters that have to be used on photon beams, include an integrated build-up cap to achieve charged particle equilibrium conditions. These features, together with the possibility to create a permanent record of the dose, make this system particularly suitable for in vivo dosimetry in treatment techniques such as brachytherapy, total body irradiation and 3-D conformal radiation therapy. Technical aspects of the design and tests of the performance of the OneDose^TM^ system in measuring dose per monitor unit in different conditions using the AAPM TG-21 protocol [23] have been described in the literature [15].

In in vivo dosimetry all those factors which influence dose deposition, especially when very high doses of radiation are prescribed, have to be taken into account. However, all these factors may not all influence simultaneously the delivery of a specific dose. In addition, even the best in vivo dosimeter cannot distinguish the causes of a dose discrepancy, but it records only their total effect. All the possible error sources need to be investigated to provide an accurate estimation of the delivered dose. The aim of this work is to use the OneDosePlus^TM^ system to investigate the dosimetric effect of the movement of patients in selected tumor sites (breast and prostate) during radiotherapy treatments.

## Methods

**Figure 1:**
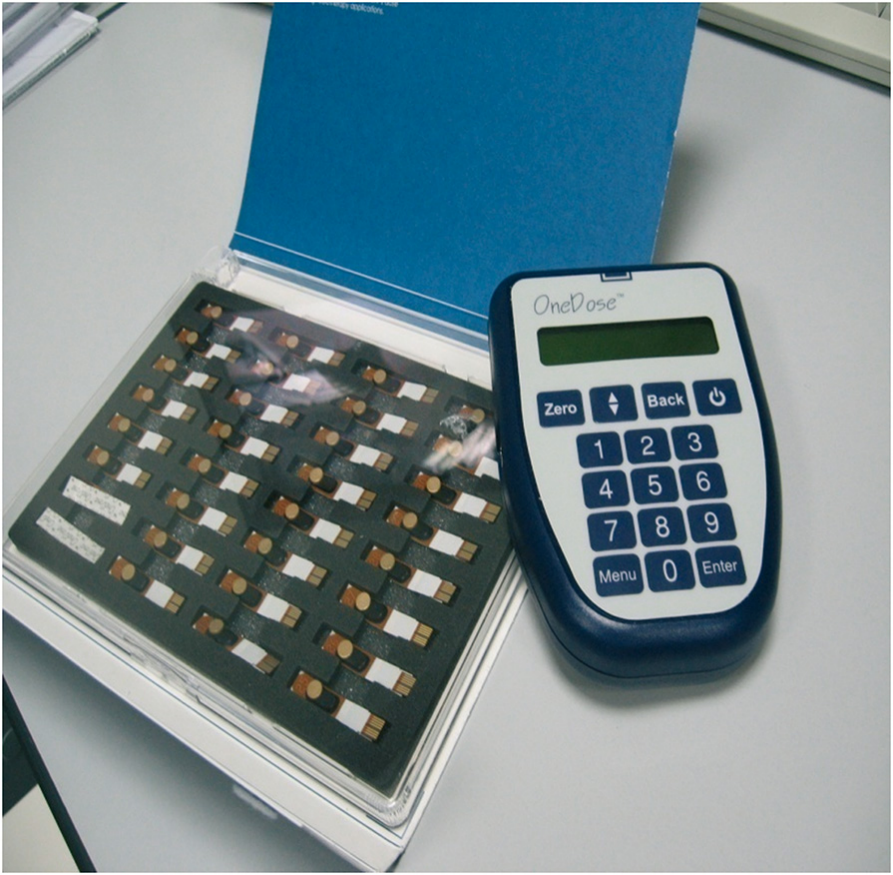
**OneDosePlus**^**TM **^**system. **The system comprises single p-type wireless MOSFET detectors and a handheld reader.

The OneDosePlus^TM^ detector system comprises a single p-type wireless MOSFET detector and a handheld reader. The manufacturer provided one reader and individual dosimeters from the same manufacturing lot (Figure 1). The MOSFET detectors have physical dimensions of 3.5 x 0.7 cm^2^ with an active area of 300 x 50 μm^2^ situated in the center of the exit surface build-up cap. They are provided with an adhesive strip to be attached to the patient’s skin. The build-up cap, instead, is a tin disk of 1.194 ± 0.008 mm thickness (equivalent to 1.4 cm in water) and 5.004 ± 0.254 mm diameter, flash coated with gold to prevent oxidation. A green dot specifies the active area. The dose measured by the detector is the maximum of the Percentage Depth Dose (PDD), and is referred to the corresponding maximum depth, d_max_ in water. For a 6 MV beam, the measurements correspond to the dose at d_max_ equal to1.4 cm. For all the other energies, correction factors relate the MOSFET readings to the corresponding doses at d_max_.

The manufacturer system specifications [14] are: dose range between 0 and 500 cGy, with an accuracy of ±5% (within 2 Standard Deviation, 2SD) and ±2 cGy for doses below 30 cGy; energy range from ^60^Co to 18 MV; SSD from 80 to 120 cm and dose linearity from 2 to 400 cGy; no angular dependence (limited to ±30° incident beam angle) and no temperature dependence (accuracy maintained to up to ±5°C variation during zeroing and postdose reading). The dosimetric system is factory calibrated with a ^60^Co beam in full build-up conditions and in the following settings (hereafter named “standard conditions”): 6MV photon beam, SSD = 100 cm, 10 cm x 10 cm field size, 300 MU/min dose rate and no wedge. To relate MOSFET readout to dose in conditions other than standard, the manufacturer provides correction factors. These factors are a function of treatment specific parameters such as energy, SSD, field size, wedge filter characteristics and dose rate (energy/modality corrective factors). They are written onto each dosimeter and specified in a calibration sheet (Lot Calibration). Calibration factors are determined for each single dosimeter while the correction factors are determined from a random sampling of detectors and applied to the entire lot. After irradiation the dose is measured by inserting the dosimeter in the handheld reader. If treatment specific parameters are entered before irradiation, the reader shows the corrected dose, otherwise, standard conditions are assumed and correction factors can later be applied by hand calculation. Optimum results are obtained if the post-dose reading is taken 2.5 minutes after irradiation to avoid fading.

Prior to implementation for in vivo dosimetry, the OneDosePlus^TM^ system has been thoroughly investigated in two phantoms, a thorax anthropomorphic phantom and a slab phantom, to establish a baseline of dose discrepancy with respect to our TPS. This part of the study was aimed at establishing an Action Threshold (AT) [5,24], defined as the maximum accepted discrepancy between the dose measured with the detector and the dose calculated with the TPS for a single field (in our procedure only one field at a time is verified). A single AT has been established, one specific for breast and one for prostate treatments, from the analysis of the tests in phantoms, and it has been used as an indicator of the correctness of breast and prostate treatments themselves. Two TPSs were used: the Precise Plan (Elekta, Crawley, United Kingdom) for breast and prostate treatments and Pinnacle^3^ version 8.0 m (Philips Medical System, Andover, MA), for prostate treatments only. Measurements were performed on two different linear accelerators: an Elekta Precise (Elekta, Crawley, United Kingdom), with nominal X-ray energies of 6 and 15 MV and with Precise Plan as TPS, and an Elekta Synergy S (Elekta, Crawley, United Kingdom), with nominal energies of 6 and 18 MV and with Pinnacle^3^ as TPS, respectively. Consequently, the AT has been determined in each combination accelerator + TPS. The measurements were performed without inserting the manufacturer’s corrective factors into the reader before treatment but correcting the readings after the treatment using the formulas in the lot calibration. This solution was chosen to minimize the duration of the procedure before treatment.

Initially the patient set-up was verified and, if needed, corrected using a 2D (breast and some prostate patients) or a 3D (only prostate patients) verification system. In the 2D verification procedure, some structures were outlined by the Radiation Oncologist both on the Digitally Reconstructed Radiographs (DRRs) and the portal images acquired by the IVIEWGT (Elekta, Crawley, United Kingdom). The displacements were evaluated after the software matched the outlines in both images. The set-up was corrected if the displacements exceeded 2 mm. In the 3D verification procedure, instead, the registration between the planning CT and the pre-treatment Cone Beam CT (CBCT) scan, was performed automatically by the XVI software (Elekta, Crawley, United Kingdom) using a 3D chamfer matching algorithm. The set-up was corrected if the calculated displacements exceeded 2 mm. Only translational set-up errors were considered and corrected online, as our treatment couch cannot perform pitch and roll rotations. However, the patient was always repositioned whenever calculated rotational set-up errors were 1° or more.

Two MOSFETs were used for each patient: one for the first and one for the last field of the treatment session (in our centre prostate treatments is delivered with six fields while breast treatments with two tangential opposing wedged fields). If the discrepancy between the measured dose, corrected with the appropriate corrective factors, and the dose calculated with the TPS was above the AT value, an intra-fraction motion error was assumed to have occurred.

### A. Phantom tests and AT calculation

To determine the AT for breast treatments, a thorax anthropomorphic phantom (model RS-111, RSD Radiology Support Devices, Inc. USA) has been used. A typical breast tangential medial wedged field (6 MV photons, asymmetrical field size, SSD 92 cm, 30° wedge, 100° collimator angle and 305° gantry angle) has been simulated with the Precise Plan as TPS, having energy, collimator angle, gantry angle, SSD, field size and wedge similar to those used for patient’s treatments. Only the tangential medial wedged field has been considered as both tangential fields (medial and lateral) are equal and opposite with the isocenter placed at the midpoint of the target in the longitudinal direction. The field was strongly asymmetrical in the wedge direction as in the majority of breast treatments. For prostate treatments, we have used a field equivalent square of 7.5 cc, with a SSD of 88 cm, an energy of 18 MV and a collimator angle of 0°. Only the 90° gantry field (rotated to 0° gantry) on a 30x30x20 cm^3^ RW3 slab phantom (ρ=1.045 g/cm^3^) has been simulated (90° and 270° gantry angles are equal and opposite with the isocenter placed at the midpoint of the target in all directions). Also in this case, the chosen set-up has been as much as possible similar to that of the majority of prostate treatments.

For each field, and for each measurement, which was repeated ten times, we defined the dose discrepancy, Δ_i_, as the percentage difference between the dose measured with the dosimetric system, Di, and D_c_, the reference dose calculated with the TPS in the same phantom set-up:

(1) $$\Delta_{i}=\frac{D_{i}-D_{c}}{D_{c}}$$

Finally, we calculated the mean dose discrepancy, indicated with Δ, and its standard deviation (Δ±SD (Δ)). Δ was used for each patient in each measurement session, to calculate the difference between the dose actually measured at the surface patient projection of the isocenter, and that calculated at the isocenter using the corresponding TPS, during in vivo check dosimetry. The AT, instead, was calculated as twice the standard deviation of the ten differences (within the confidence interval of 95%). Other plausible combinations of SSD, gantry angle, field size and wedge angle have been randomly considered and the differences with the corresponding calculated doses evaluated. This set of measurements has led to an additional error of 0.5% to add to the AT previously calculated. A further 0.5% has been added to take into account the MOSFET positioning error due to difficulty to exactly place the detector at the surface projection of the isocenter on the phantom, or on the patient. In our tests, the SD was considered as the random error; while Δ as the systematic error. The latter has not been taken into account for in vivo evaluations since it was found to be negligible.

To investigate the most likely directions of the intra-fraction patient movements, five MOSFETs were placed at fixed displacements with respect to the beam central axis (surface projection of the isocenter on the phantom), considered as the reference point, and irradiated (we carried out one displacement at a time). We wanted to determine not only the direction of the displacements during each treatment session but also if the system was actually able to detect them. We first considered 3 mm (the displacement that does not affect dose target distribution) in all 3D directions, then 1 cm. In breast treatments and towards the wedge toe, the displacement performed was 0.7 cm, since 1 cm displacement would lead to the field edge. The directions of displacements were (looking towards the gantry): couch to the right, couch to the left, couch to gun, couch to target, couch up and down. The average values for each direction and displacement length, have been put in comparison to the reference. A statistical analysis of these data has been performed to establish the sensitivity of the system in detecting the displacements.

### B. In vivo measurements

Twenty five patients with breast cancer and twenty five patients with prostate cancer were considered for this study. Each dosimeter was attached to the patient’s skin with its build-up cap area at the surface projection of the isocenter and as perpendicular as possible to the beam central axis. The approximate perpendicularity (within ±30°) between the beam axis and MOSFET surface was verified by the Radiation Technologist with a rigid sheet of paper placed on the MOSFET surface. The patients with breast cancer had their treatments planned with the Precise Plan TPS and were irradiated with the Elekta Precise accelerator. Patients with prostate cancer, were split into two groups: the first group included 10 patients treated with the same accelerator, the same TPS and the same 2D verification system as the breast patients; the second group, instead, included 15 patients whose treatments were planned with Pinnacle^3^ and who were irradiated with the Elekta Synergy S. In both TPSs, the doses were calculated isolating the contribution of the field which was to be measured with the MOSFET. The calculated dose value was taken at the depth d_max_ along the central ray passing through the isocenter. These doses were compared with the measurements obtained with the dosimetric system, after correction by energy/modality corrective factors. For each patient, the measurements were performed, once a week, using one MOSFET for the selected field of the treatment plan.

**Figure 2:**
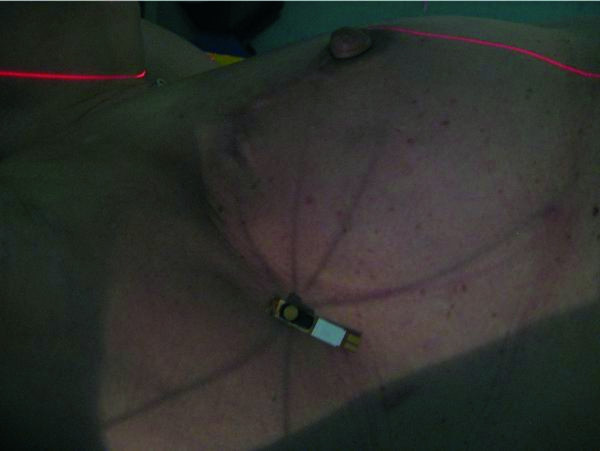
**Breast treatment. **MOSFET detector positioned at the surface projection of the isocenter on a patient treated for breast cancer (lateral field).

In patients with breast cancer, both medial and lateral fields were monitored. Figure 2 shows the MOSFET detector positioned at the surface projection of the isocenter for the lateral field; the approximate perpendicular beam incidence is also visible. To correct the set-up, the IVIEWGT verification system has been used before each irradiation. A commercial breast board (AKTINA Medical) was used as immobilization device. The dose fractionation schedule was 4400 cGy delivered to the isocenter in 16 fractions (275 cGy/fr) with 6 MV photons. One detector was irradiated for each field, once a week. This amounted to four detectors per field at the end of the treatment course. The eight dose values measured along the entire treatment were averaged and the resulting value together with its standard deviation, has been reported and compared to the dose calculated with the TPS.

**Figure 3:**
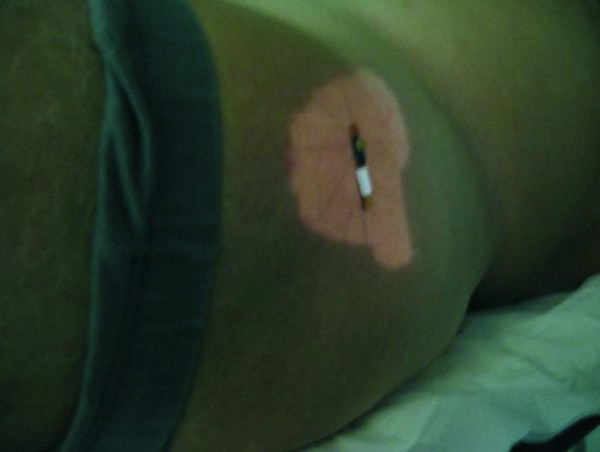
**Prostate treatment. **MOSFET detector positioned at the surface projection of the isocenter on a patient treated for prostate cancer (90° field).

In patients with prostate cancer, the treatment field at 90°gantry angle was first verified, then the field at 270°, which was the last field delivered in each treatment session (the treatment being delivered using six fields at 90°, 45°, 135°, 225°, 315°, 270°) (Figure 3). The treatment was delivered in 33 sessions over 6 weeks with a total dose of 6600 cGy (200 cGy/fr at the isocenter). The energy used was 15 MV for the Elekta Precise and 18 MV for the Elekta Synergy S. A home-made foot block and a pillow under the head were used as immobilization device. Twelve dosimeters (six for 90° gantry angle and six for 270° gantry angle) for each patient have been irradiated at the end of the treatment course. The dosimeters were placed on the hip as perpendicular as possible to the beam central axis. The twelve dose values measured along the entire treatment were averaged and the result, together with its standard deviation, has been reported and compared to the reference dose calculated with the corresponding TPS. The attenuation of the MOSFET detectors was not considered in treatment planning since it was measured and found negligible according to our in vivo procedure. Written informed consent was obtained from the patient for publication of this report and any accompanying images.

## Results

### A. Phantom tests and AT calculation

For the tangential medial wedged field, the average dose of ten repeated measurements on the Elekta Precise was (379.0 ± 5.3) cGy, while the corresponding dose calculated with Precise Plan TPS (TPS1) was 379.7 cGy. The SD of the ten dose differences was about 1.5%. The same result has been obtained for the prostate treatment, using the same linear accelerator and TPS (TPS1) of the breast treatment (not reported). Therefore, AT1 = 5.0%. For prostate treatments and for the Elekta Synergy S the average dose of ten repeated measurements was (194.4 ± 2.3) cGy, while the corresponding dose calculated with Pinnacle^3^ TPS (TPS2), was 193.5 cGy. The SD was 1.2%. Making the same considerations above reported, the overall result for AT2 was ± 4.5%.

The results of the displacements made in the phantoms with respect to the reference point, are reported in Additional file 1: Table S1 and Additional file 2: Table S2 for breast and prostate treatments, respectively. For each displacement, we have assumed that the values measured followed the Student’s t distribution with ν = 5 degrees of freedom. From the statistical analysis of the data in Additional file 1: Table S1, we have found that, for each displacement, the differences between the average values and the corresponding reference, are statistically significant only in three cases (p value <0.005). The system, therefore, is sensitive to displacements of: 1) 0.7 cm towards the toe of the wedged field (named s1), which corresponds to a displacement towards the penumbra region (the worse value); 2) 1 cm toward the heel of the wedged field, with the couch to the left (named s2) and 3) 1 cm with the couch up (named s3). The latter is the displacement that can be due to the respiratory acts of the patient where a large SSD variation can be observed. Shaded rows mark these displacements. The maximum discrepancies found were −6.0%, -4.9% and +3.5% for s1, s2 and s3, respectively. These values were smaller than AT1 (±5.0%), except s1. If we consider 2 SD, the new value of s1 becomes smaller than AT1. Therefore, the system is not able to detect displacements smaller than 1 cm.

For prostate treatments, the corresponding statistical analysis of data reported in Additional file 2: Table S2, has shown that the differences between the reference dose value and the average dose for each displacement, are statistically significant in two cases (197.9 ± 2.1 cGy and 190.3 ± 1.9 cGy, respectively) which corresponds to displacements of the couch up and down. Shaded rows mark these displacements. The maximum discrepancy found was −2.6% and +3.3%, both smaller than AT2. Also in this case, the system can detect only displacement not smaller than 1 cm. For prostate data and for TPS1 + Elekta Precise accelerator, similar results have been obtained (not shown).

### B. In vivo measurements

**Figure 4:**
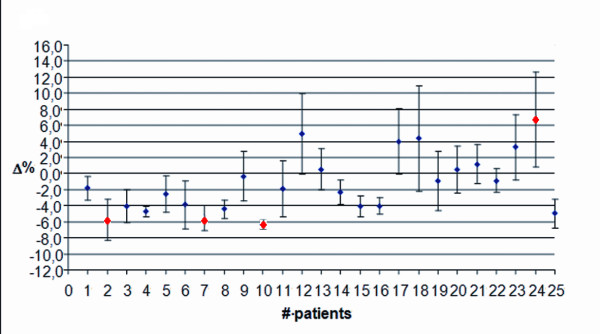
**Breast treatment with the Elekta Precise. **Average discrepancy using TPS1 and a 2D verification system, for both medial and lateral fields along the entire treatment course.

**Figure 5:**
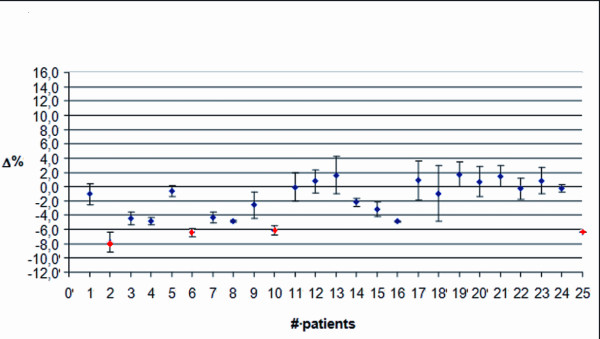
**Breast treatment with the Elekta Precise. **Average discrepancy using TPS1 and a 2D verification system, for the medial field along the entire treatment course.

**Figure 6:**
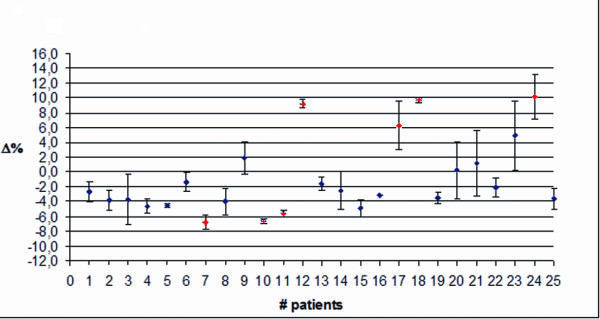
**Breast treatment with the Elekta Precise. **Average discrepancy using TPS1 and a 2D verification system, for the lateral field along the entire treatment course.

Figure 4 shows the data for twenty five patients with breast cancer and TPS1. The mean discrepancy was −1.4% with a SD of 3.8%. These values were averaged on both fields (medial and lateral) and along the entire treatment. To investigate if the patients moved during both fields or only during the last field of the treatment session, data were also split into medial and lateral field only. Figures 5 and 6 show the average discrepancy for the medial and the lateral field, respectively. In these plots, the mean discrepancy was −2.2% with a SD of 3.0%, and 0.9% with a SD of 5.1% for the medial and the lateral field, respectively. The lateral field show a wider data dispersion with respect to the medial one. In the figures, red dots mark the Δ values exceeding AT1.

**Figure 7:**
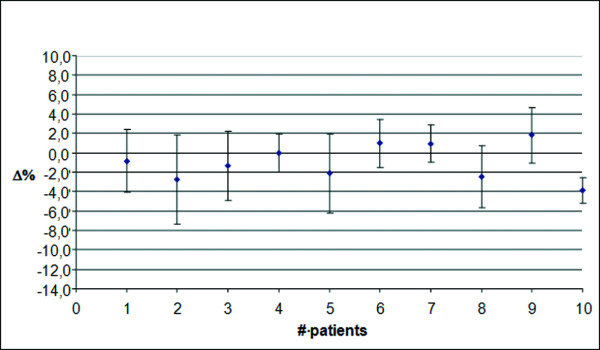
**Prostate treatment with the Elekta Precise. **Average discrepancy using TPS1 and a 2D verification system along the entire treatment course.

In Figure 7, data for prostate patients using TPS1 and a 2D verification system, are shown. The discrepancies reported are lower than those observed in breast patients, with a mean discrepancy of −1.0% and a SD of 1.9%. It has to be pointed out that only average values have been reported. Only for two patients and only for one treatment session, the discrepancy exceeded AT1. This happened in the first treatment session and never again.

**Figure 8:**
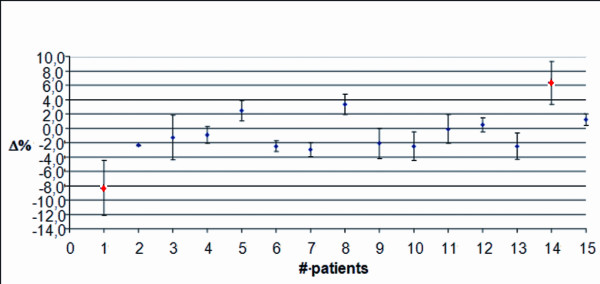
**Prostate treatment with the Elekta Synergy S. **Average discrepancy using TPS2 and a 3D-CBCT verification system along the entire treatment course.

In Figure 8, data for prostate patients using TPS2 and a 3D-CBCT verification system, are displayed. The mean discrepancy was −1.6% with a SD of 3.0%. Red dots mark the patients whose Δ values have been found to exceed AT2 in each treatment session. The first one, had a Δ value negative, indicating a displacement towards the source. The other, had a positive discrepancy indicating that the patient moves away from the source. A wider data dispersion, however, can be observed in the patients treated with Elekta Synergy S (SD = 3.0%) compared to those treated with Elekta Precise (SD = 1.9%). In the statistical analysis of patients treated with Elekta Synergy S, except for patients # 1 and 14 (that can be considered as “outliers”), the data dispersion is similar (SD = 2.0%) to that obtained for patients treated with Elekta Precise (SD = 1.9%), with a similar mean discrepancy too (−0.8% compared to −1.0%). Finally, it can be observed that, again except for patients # 1 and 14, the data dispersion for each patient is less wide than that obtained in patients treated with Elekta Precise, shown in Figure 7.

## Discussion

The AT values calculated using data from phantom tests for two different TPSs, were found to be very similar, ±5.0% for TPS1 and ±4.4% for TPS2, and of the same order of the magnitude of the intrinsic system uncertainty (2SD = ±5.0%). This is due to the fact that SD1 is not so much different from SD2, even if SD2 has been determined from TPS2 (Pinnacle^3^) which is more accurate than TPS1 (Precise Plan) in calculating doses (SD1 is 1.5% for TPS1, and SD2 is 1.2% for TPS2). This means that the system is reproducible with respect to each TPS. This result is similar to those found in other institutions [5,24] using other measurement devices like different MOSFET or diode based dosimeters.

Among the twenty five patients treated for breast cancer, only in four of them Δ was larger than AT1 in each measurement session over a treatment lasting two weeks and half. Other six patients, randomly moved during the treatment. After the first session, we told the patient not to move during the treatment but the results of the measurements of the subsequent weeks reported in our figures, show that they didn’t follow our instructions or, more likely, the position of treatment was too hard to keep for the long treatment time (it lasted about 10 minutes), so they probably relaxed their arms causing a shift of the point in which the dosimeter was placed. This shift could have caused a change of the measured dose due to the gradient across the wedge and also across the penumbra of the field.

The discrepancies larger that the corresponding ATs, have a general negative trend. However, some of them have positive values indicating that either the corresponding fields are different from that simulated in the phantom study (likely in patient # 12, where the length of the field was larger than 1 cm in the toe direction of the wedge); or that an abdomen expansion due to a deeper than average inspiration had happened (likely in the patient # 24, who had a discrepancy value larger then AT1, both in medial and lateral fields).

In vivo results for patients with prostate cancer have shown better results compared to patients with breast cancer. This result indicates that this kind of treatment is more reproducible, also due to the immobilization system which makes movements difficult. Patients # 1 and 14 have been positioned with a 3D-CBCT scanner, known to be more accurate than a 2D system [25-27]. This clearly shows that even if the patient is positioned using the best and the most accurate verification system, motion during the treatment session causes a loss of accuracy in the treatment. However, the general trend of a reduced data dispersion, calculated along the entire treatment, for patients treated with Elekta Synergy S compared to those calculated with Elekta Precise, indicate that if patients don’t move and they are positioned using the best 3D-CBCT scanner, treatment accuracy improves.

In Image-Guided Radiation Therapy (IGRT) and Intensity Modulated Radiation Therapy (IMRT) techniques, the time of patient setup and treatment delivery has increased, together with the need to monitor and manage patient motion during treatment. There are different systems based on new technologies to monitor internal tumor motion, patient motion or both during treatment. As concerns external motion detection and measurement, Infrared (IR) cameras which use infrared external markers [28,29], spirometry [30], electromagnetic positioning systems [31] or in-house devices [32,33] have been used to monitor patient’s and respiratory motion. The accuracy declared by the manufacturers in detecting displacements of the chest wall is about 1 mm for the commercial devices, and increases to 0.5 - 1.25 cm in the in-house systems. Some of these systems focus on detecting chest wall motion to predict tumor motion, the commercial systems being, in addition, quite expensive. The external displacements can however differ from internal tumor displacements up to about 2 cm, as reported by some authors [34-36]. Tumor motion, can be directly measured by surgically implanting fiducial markers into the tumor. The movements of these markers can be then tracked using fluoroscopy [37] or other techniques [38]. The use of internal markers, suffers from high level of morbidity (the clips can migrate) and, when fluoroscopy is used to track the markers, additional dose is delivered to the patient [35,39]. All these systems concentrate in extracting information about intra-fraction organ motion but they not consider the dose that the target really receives. In a recent publication [32], the authors use a new 4D in vivo dosimetry tool (based on MOSFET dosimeters combined to an electromagnetic positioning sensor) to simultaneously measure real time dose delivery to the center of the field and surface lung motion. Our methodology is based on the idea of using only *“*in vivo*”* dosimetric information to detect intra-fraction patient movements during treatment. It is very simple to implement, can be used in every type of treatment, it is not expensive and can 1) give both dose measurements and intra-fraction patient movements; 2) use in vivo dose measurements to give information about the direction of target movements and 3) estimate the dose received from the isocenter (inside the target) from the dose measured in two opposing fields. The main limitations, however, are that the system can only monitor external motion and that the assessment of the intra-fraction motion cannot be made on-line in a continuous fashion but only at the end of the treatment session.

The accuracy of the method was found to be of 1 cm which means that displacements larger than 1 cm can be detected. However, this depends on whether a displacement produces a significant difference between measured and calculated dose, which in turn depends on the kind of treatment and treatment site. Our work was focused on 3D conformal treatments; due to extreme sensitivity of the system to rapid dose variations, we believe that the accuracy can be improved when monitoring movements in patients treated with IMRT.

## Conclusion

The dosimetric performance of the OneDosePlus^TM^ system for in vivo dosimetry has been studied with respect to two different TPSs. The ATs established in phantom tests have been found to be of the same order of magnitude and independent from the TPS used. This procedure applied to patients after correction of the set-up errors, has allowed the identification of large random movements in some patients, which resulted to be more pronounced in breast than in prostate treatments. In addition, the results obtained indicate that these movements are not dependent on the kind of the verification system (2D or 3D) used to correct the set-up. The method decribed in the paper, easily implemented in clinical practice, combines all the advantages of the OneDosePlus^TM^ system, such as the small size of the dosimeter, absence of cables, instant read-out, permanent storage of dose and ease of use, with the possibility of detecting intra-fraction patient movements.

## Competing interests

The authors have no financial disclosures or conflicts of interest to report.

## Authors’ contributions

MDF reviewed and analyzed the data, performed statistical analysis, created the figures, and drafted the manuscript. MD reviewed and analyzed the data, critically revising the study and assisted in drafting the manuscript. ALB, MR participated in the design of study, interpretation and analysis of data. EP, BT were responsible in positioning the detectors and collected dosimetric data. GT, RB, LDM participated in the study design and coordination and helped to draft the manuscript RS provided significant intellectual contribution and reviewed the manuscript All authors read and approved the final manuscript.

## Supplementary Material

Additional file 1 **Table S1. **Comparison between ${\bar{D}}_{m}$ and the average doses measured simulating fixed displacements for a tangential breast field. Click here for file

Additional file 2 **Table S2. **Comparison between ${\bar{D}}_{m}$and the average doses measured simulating fixed displacements for a 90° prostate field projected to 0°. Click here for file
