# Supplementary material for: Is the in vivo dosimetry with the OneDosePlusTM system able to detect intra-fraction motion? A retrospective analysis of in vivo data from breast and prostate patients
Source: Radiat Oncol. 2012 Jun 20;7:97. doi: 10.1186/1748-717X-7-97 (PMC3526469; doi:10.1186/1748-717X-7-97)
Supplement: Additional file 1 — Table S1. Comparison between D¯m and the average doses measured simulating fixed displacements for a tangential breast field. [file 1748-717X-7-97-S1.doc]

**Table S1**

| **Energy**  **(MV)** | **Displacement** | $\bar{D}_{m}$**±SD**  **(cGy)** |
| --- | --- | --- |
| 6 | central | 379.0± 5.3 |
|  | 3 mm left | 377.1± 5.2 |
|  | 3 mm right | 377.3± 5.5 |
|  | 3 mm down | 375.8± 5.1 |
|  | 3 mm up | 380.9± 5.1 |
|  | 3 mm gun | 375.3± 5.7 |
|  | 3 mm target | 374.9± 5.9 |
|  | 1.0 cm left | 366.0±5.0 |
|  | 0.7 cm right | 362.1 ±5.1 |
|  | 1 cm down | 373.2±5.8 |
|  | 1 cm up | 387.0± 5.0 |
|  | 1 cm gun | 373.7±5.5 |
|  | 1 cm target | 378.7 ±5.4 |
