# Supplementary material for: Is the in vivo dosimetry with the OneDosePlusTM system able to detect intra-fraction motion? A retrospective analysis of in vivo data from breast and prostate patients
Source: Radiat Oncol. 2012 Jun 20;7:97. doi: 10.1186/1748-717X-7-97 (PMC3526469; doi:10.1186/1748-717X-7-97)
Supplement: Additional file 2 — Table S2. Comparison between D¯mand the average doses measured simulating fixed displacements for a 90° prostate field projected to 0°. [file 1748-717X-7-97-S2.doc]

**Table S2**

| **Energy**  **(MV)** | **Displacement** | $\bar{D}_{m}$**±SD**  **(cGy)** |
| --- | --- | --- |
| 18 | central | 194.4± 2.3 |
|  | 3 mm left | 193.2± 2.1 |
|  | 3 mm right | 192.8±2.4 |
|  | 3 mm down | 193.0 ±1.7 |
|  | 3 mm up | 194.8±2.0 |
|  | 3 mm gun | 193.1±2.1 |
|  | 3 mm target | 193.8 ±1.9 |
|  | 1 cm left | 192.0± 2.2 |
|  | 1 cm right | 197.3±2.4 |
|  | 1 cm down | 190.3 ±1.9 |
|  | 1 cm up | 197.9±2.1 |
|  | 1 cm gun | 192.6±209 |
|  | 1 cm target | 192.9±2.4 |
